# Supplementary material for: Transcriptomics Integrated with Metabolomics Reveals the Effect of Cluster Thinning on Monoterpene Biosynthesis in ‘Muscat Hamburg’ Grape
Source: Foods. 2021 Nov 6;10(11):2718. doi: 10.3390/foods10112718 (PMC8623782; doi:10.3390/foods10112718)
Supplement: Supplementary file 1 [file foods-10-02718-s001.zip › foods-1437200-supplementary.pdf]

Supplementary Figure S1. Technological parameters of grape berries from different loading grapes during the ripening stages. Data are presented as mean ± standard error (n=3). Different letters indicate significant differences (Duncan' s multiple range test, $p \leq 0.05$).

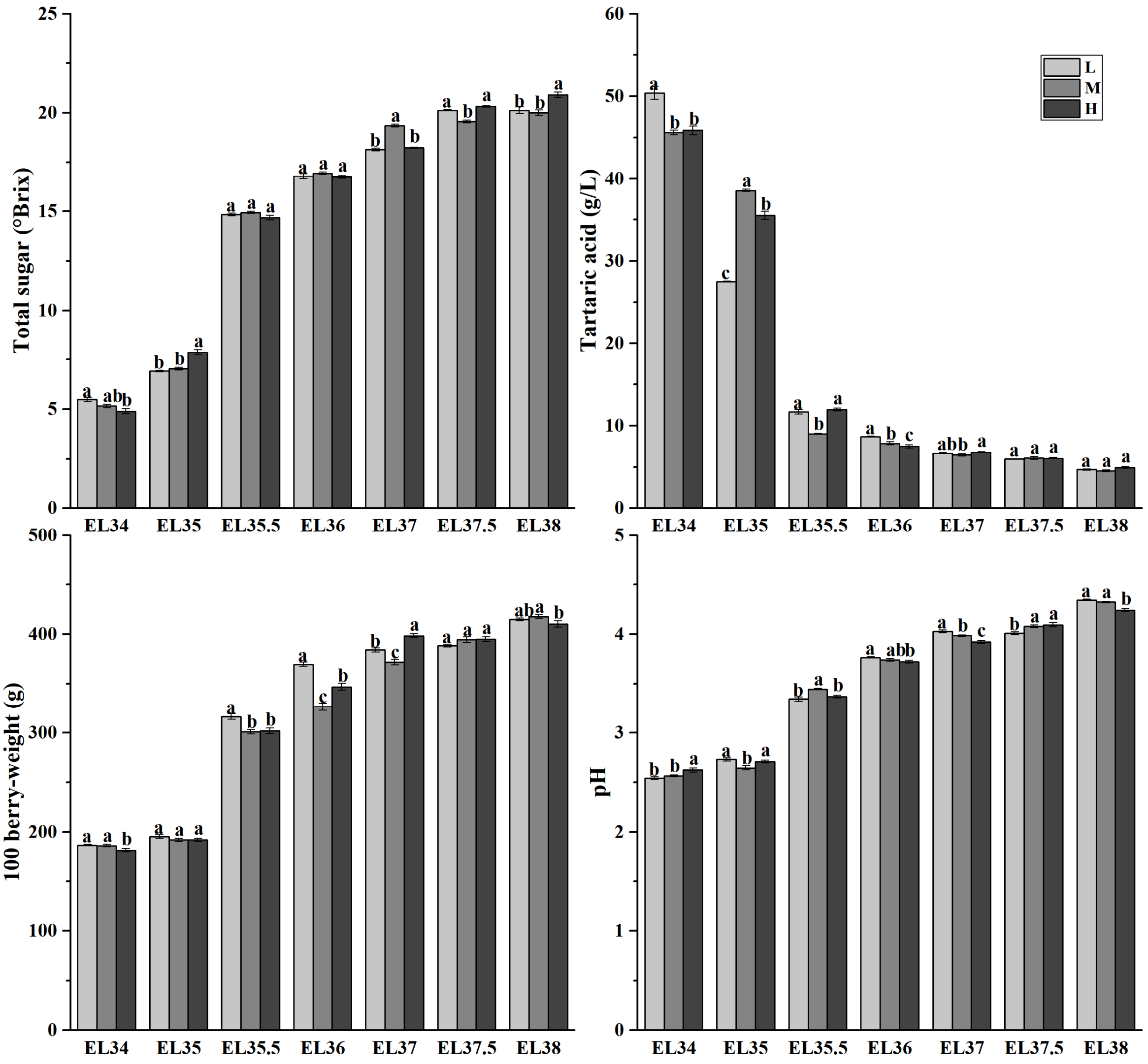

Supplementary Figure S2. Changes of total monoterpenes (total free and total bound) profiles in Muscat Hamburg grape at six developmental stages.

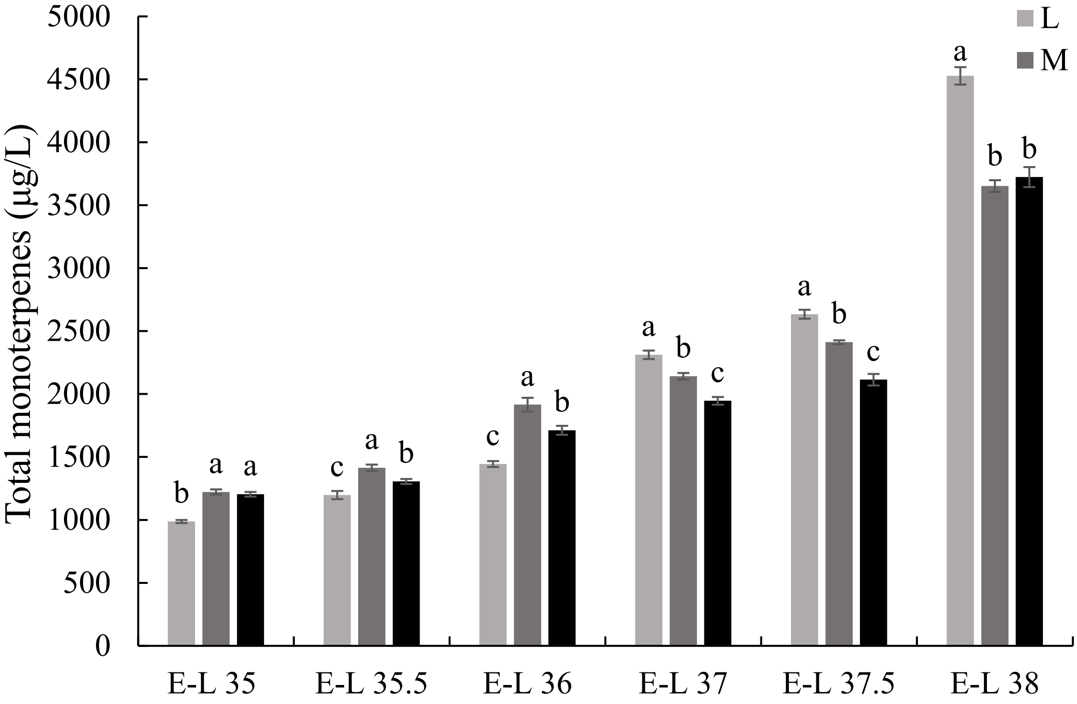

Supplementary Figure S3. eggNOG function classification on brown module genes.

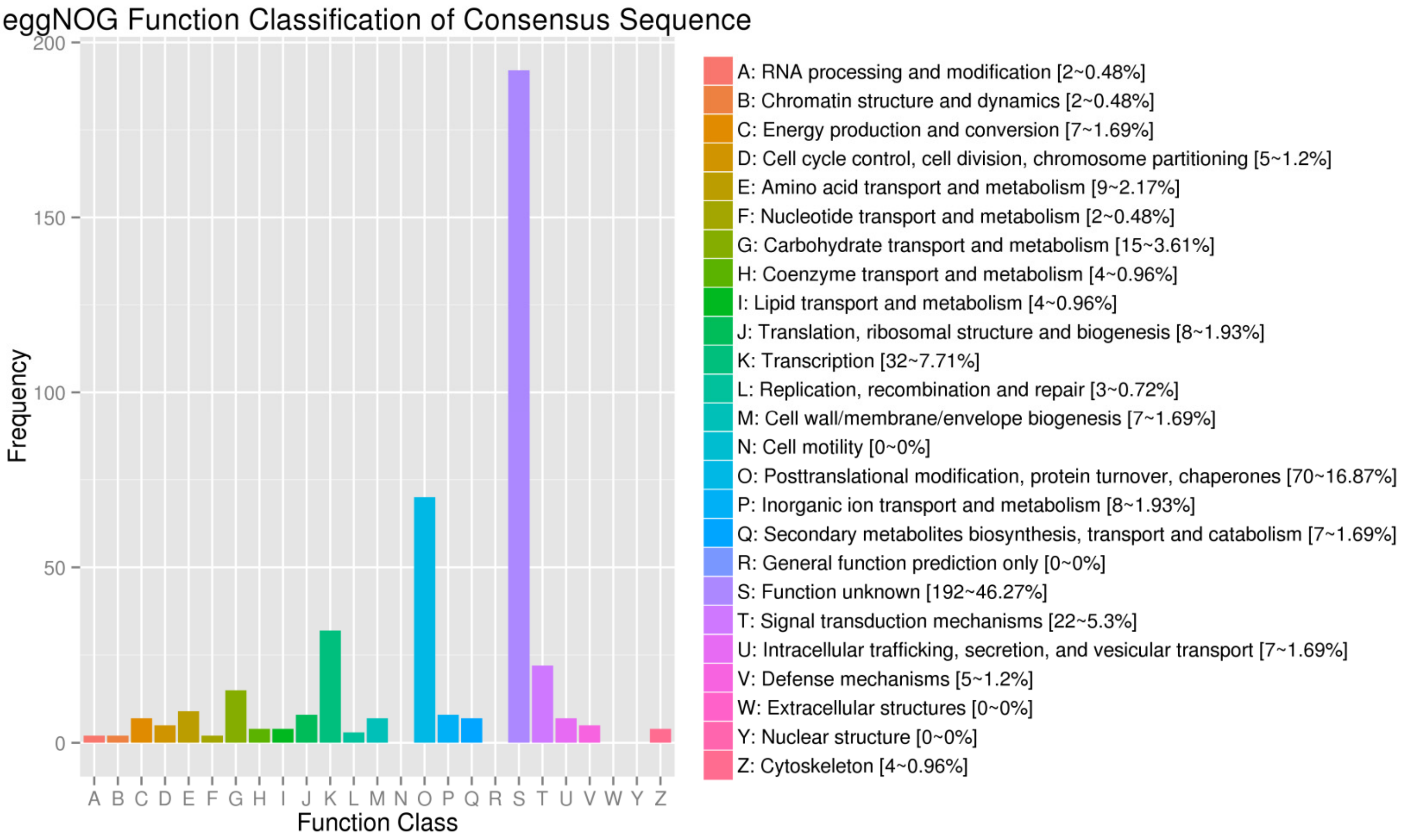

Supplementary Figure S4. KEGG analysis on brown module genes. Column length indicates the number of genes under this category.

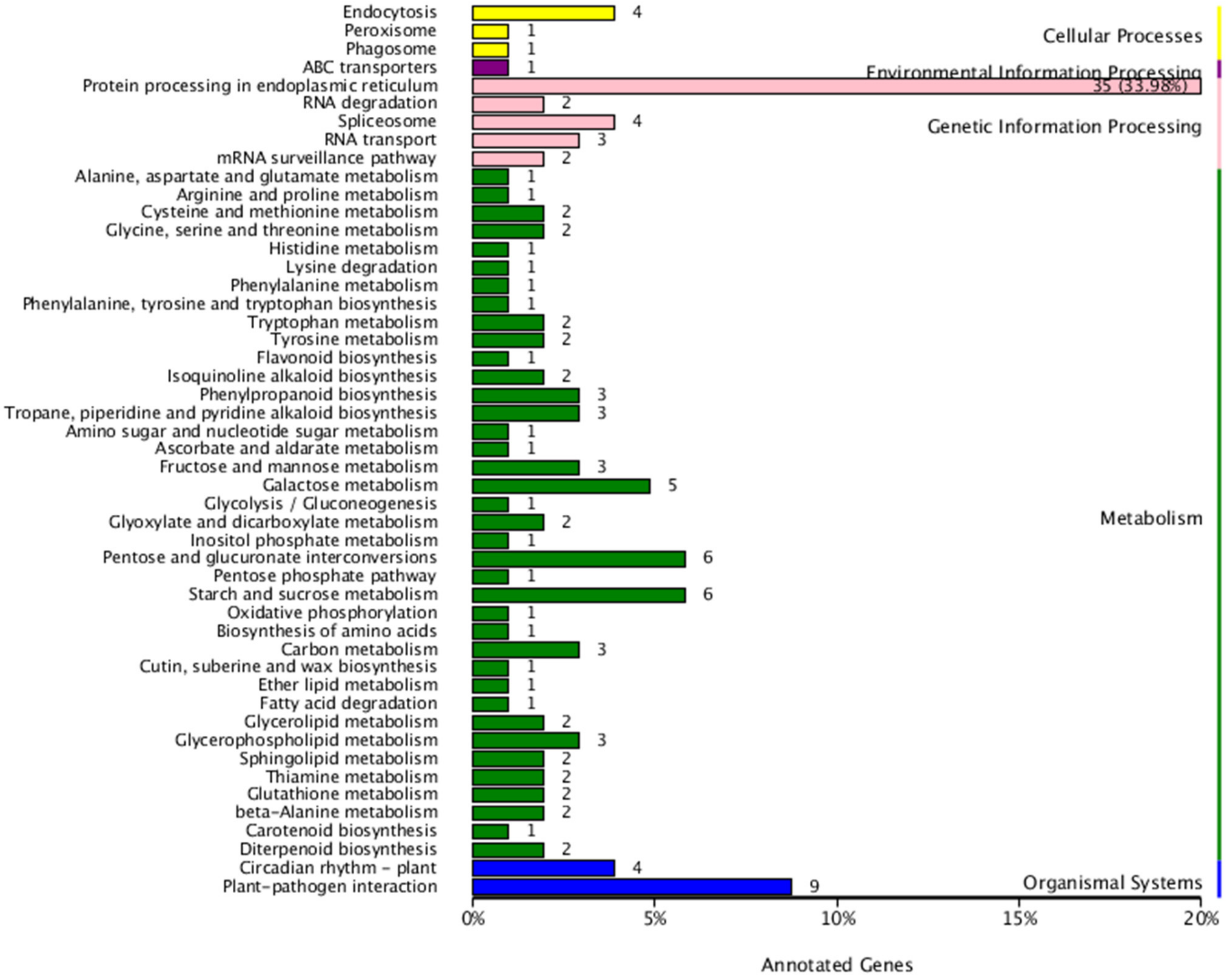

Table S1. Changes of free monoterpenes profiles in Muscat Hamburg grape at six developmental stages.

Free monoterpenes (μg/L)	E-L 35			E-L 35.5			E-L 36		
	L	M	H	L	M	H	L	M	H
β-Myrcene	2.71±0.01b	2.86±0.08a	2.95±0.04a	5.89±0.22a	4.65±0.12b	3.75±0.04c	9.51±0.97c	15.96±0.05a	13.56±1.19b
Limonene	0.63±0.03c	1.18±0.04b	1.34±0.06a	2.48±0.18c	3.00±0.02a	2.79±0.03b	5.32±0.04c	13.64±0.27a	10.59±0.03b
Phellandrene	0.48±0.02c	0.61±0.01b	0.68±0.01a	1.00±0.04a	0.85±0.01b	0.82±0.01b	1.82±0.01c	2.93±0.01a	2.63±0.04b
β-trans-Ocimene	2.48±0.01c	2.52±0.00b	2.55±0.01a	3.55±0.11a	3.30±0.03b	3.04±0.02c	5.31±0.04c	8.21±0.01a	7.74±0.02b
γ-Terpinen	1.30±0.04c	1.99±0.01b	2.13±0.09a	1.09±0.02c	1.32±0.01b	1.71±0.02a	1.18±0.01c	1.90±0.01a	1.68±0.01b
β-cis-Ocimene	2.52±0.01c	2.58±0.02b	2.65±0.03a	4.10±0.16a	3.78±0.03b	3.47±0.01c	6.78±0.01c	11.55±0.05a	10.67±0.07b
Terpinolen	1.04±0.02c	1.28±0.01b	1.36±0.03a	1.22±0.03c	1.59±0.01b	1.69±0.02a	1.67±0.04c	4.46±0.08a	3.55±0.03b
Cis rose oxide	1.08±0.01a	1.08±0.02a	1.08±0.01a	1.17±0.01a	1.13±0.02b	1.12±0.01b	1.34±0.02a	1.30±0.01b	1.35±0.01a
Trans rose oxide	nd	nd	nd	nd	nd	nd	0.46±0.02a	0.08±0.01c	0.24±0.01b
Allo-Ocimene	2.41±0.01c	2.47±0.01b	2.50±0.02a	2.65±0.03a	2.68±0.01a	2.67±0.01a	3.05±0.02c	4.00±0.04a	3.75±0.02b
(E,Z) Allo-Ocimene	2.39±0.01b	2.45±0.03a	2.46±0.02a	2.52±0.02a	2.53±0.01a	2.49±0.01b	2.78±0.01c	3.16±0.03a	3.04±0.02b
Cis-furan linalool oxide	0.12±0.01c	0.34±0.02b	0.57±0.03a	0.55±0.02b	0.68±0.02a	0.68±0.01a	0.85±0.01c	1.71±0.08a	1.34±0.01b
Trans-furan linalool oxide	0.18±0.01b	0.16±0.01c	0.27±0.01a	0.68±0.02a	0.49±0.01b	0.38±0.01c	1.31±0.01c	1.79±0.04a	1.46±0.03b
Nerol oxide	13.33±0.06b	12.83±0.01c	14.09±0.04a	18.90±0.28b	20.47±0.01a	20.69±0.12a	22.29±0.29c	33.05±0.54a	30.41±0.30b
Citronellal	1.47±0.03a	1.73±0.01a	1.69±0.29a	1.61±0.01b	1.75±0.04a	1.79±0.01a	1.61±0.04b	2.17±0.02a	2.03±0.17a
Linalool	3.40±0.05a	3.26±0.03b	3.22±0.02b	26.03±0.29a	12.67±0.37b	7.95±0.11c	59.65±0.74b	68.03±0.38a	69.08±0.57a
4-Terpineol	1.53±0.03b	2.36±0.01a	2.37±0.01a	1.43±0.01c	1.64±0.02b	1.81±0.02a	1.45±0.01c	1.97±0.01a	1.78±0.04b
Neral	1.06±0.04a	1.07±0.01a	1.09±0.04a	1.13±0.02a	1.08±0.03c	1.11±0.01b	1.10±0.02b	1.29±0.05ab	1.32±0.17a
α-Terpineol	7.50±0.07c	9.78±0.03b	10.59±0.07a	9.89±0.34b	15.34±0.22a	15.66±0.45a	14.10±0.61c	47.55±1.30a	35.88±0.45b
Geranial	1.12±0.01b	1.10±0.01c	1.15±0.01a	1.50±0.02a	1.40±0.05b	1.37±0.05b	1.57±0.02b	1.87±0.02a	1.97±0.10a
β-Citronellol	1.76±0.01a	1.73±0.01a	1.79±0.11a	1.92±0.02a	1.89±0.02b	1.82±0.01c	1.96±0.01b	1.96±0.01b	2.08±0.02a
γ-geraniol	5.25±0.01a	5.25±0.01a	5.26±0.01a	5.26±0.01a	5.25±0.01a	5.25±0.02a	5.25±0.02a	5.26±0.01a	5.27±0.01a
Nerol	10.06±0.01a	10.05±0.02a	10.06±0.01a	10.57±0.02a	10.32±0.02b	10.20±0.01c	10.71±0.03c	11.68±0.06a	11.56±0.04b

Cis-isogeraniol	nd	0.61±0.02b	1.09±0.19a	0.34±0.06c	1.19±0.18b	1.66±0.08a	0.32±0.06b	1.41±0.08a	1.41±0.26a
Trans-isogeraniol	nd	0.87±0.03b	1.19±0.15a	0.38±0.06b	1.66±0.13a	1.79±0.18a	0.31±0.01c	1.86±0.03a	1.54±0.08b
Geraniol	38.01±0.02a	37.28±0.03b	37.34±0.06b	45.25±0.33a	43.49±0.26b	39.69±0.12c	45.92±0.39b	56.87±0.44a	56.42±0.23a
Geranic acid	37.05±0.01c	37.18±0.05b	37.34±0.01a	38.36±0.04b	39.51±0.05a	37.73±0.01c	38.00±0.12b	38.07±0.06b	39.06±0.20a
Total	138.86±0.45c	144.60±0.33b	148.92±0.48a	189.47±2.23a	183.65±1.24b	173.13±0.62c	245.61±0.65c	343.72±2.44a	321.40±2.00b

Data are presented as mean ± standard error (n=3). Different letters indicate significant differences (Duncan's multiple range test, $p \leq 0.05$); nd represents not detected.

Continued Table S1.

Free monoterpenes (μg/L)	E-L 37			E-L 37.5			E-L 38		
	L	M	H	L	M	H	L	M	H
β-Myrcene	23.27±0.57b	26.63±1.56a	22.78±0.16b	38.06±1.76a	31.24±0.19c	35.33±0.61b	132.61±5.82c	162.29±2.81a	150.89±3.04b
Limonene	13.13±0.22c	22.78±1.24a	15.68±0.07b	26.83±0.62a	26.26±0.43a	18.63±0.03b	80.12±2.70c	100.83±2.22a	85.20±2.28b
Phellandrene	3.61±0.08b	4.81±0.05a	3.63±0.02b	6.28±0.25a	5.45±0.08b	5.30±0.08b	13.26±0.24c	17.83±0.08a	15.97±0.37b
β-trans-Ocimene	10.48±0.20b	13.08±0.50a	10.98±0.02b	17.13±1.33a	15.24±0.01b	15.93±0.15ab	60.25±3.44c	74.26±3.18a	66.60±2.54b
γ-Terpinen	1.57±0.01c	2.34±0.08a	1.76±0.02b	2.18±0.04b	2.34±0.01a	1.88±0.02c	4.98±0.22b	6.26±0.29a	5.03±0.06b
β-cis-Ocimene	14.57±0.36c	19.40±0.47a	15.80±0.06b	24.47±1.40a	22.52±0.16b	22.66±0.23b	99.36±4.92b	132.63±13.18a	116.1±3.99ab
Terpinolen	3.30±0.07c	6.69±0.27a	4.77±0.02b	6.77±0.05b	7.80±0.03a	4.66±0.08c	21.50±0.30b	26.92±0.35a	20.55±0.31c
Cis rose oxide	1.43±0.01a	1.41±0.02a	1.38±0.02b	1.52±0.03b	1.41±0.01c	1.95±0.01a	2.00±0.03c	2.80±0.06b	2.93±0.05a
Trans rose oxide	0.66±0.03a	0.19±0.01c	0.34±0.01b	0.65±0.07b	0.28±0.01c	1.88±0.05a	1.54±0.09c	2.98±0.08b	3.52±0.12a
Allo-Ocimene	4.16±0.06c	5.40±0.15a	4.58±0.02b	5.79±0.03a	5.72±0.01b	5.08±0.01c	21.13±0.71c	25.51±0.25a	22.79±0.37b
(E,Z) Allo-Ocimene	3.49±0.06b	3.79±0.05a	3.46±0.01b	4.34±0.13a	4.02±0.07b	4.02±0.02b	10.07±0.53b	12.41±0.71a	10.78±0.45b
Cis-furan linalool oxide	2.47±0.01c	3.29±0.11a	2.15±0.01b	4.74±0.10a	3.84±0.01b	3.76±0.03b	4.11±0.01c	4.48±0.02b	4.53±0.03a
Trans-furan linalool oxide	1.84±0.01b	1.96±0.02a	1.33±0.04c	2.22±0.05a	1.80±0.01b	1.85±0.01b	2.89±0.02b	3.18±0.02a	3.16±0.01a
Nerol oxide	23.71±0.04c	38.51±2.28a	26.97±0.12b	29.78±0.25b	33.35±0.11a	26.91±0.06c	53.62±0.16c	88.37±0.66a	71.00±0.47b
Citronellal	1.91±0.01b	2.88±0.05a	2.02±0.09b	2.34±0.06b	2.60±0.04a	2.23±0.06b	2.41±0.01c	3.06±0.01a	2.65±0.01b
Linalool	132.49±3.09a	118.33±4.21b	119.39±0.29b	203.89±4.76b	140.53±0.69c	210.7±2.86a	338.42±3.64b	395.4±14.85a	406.72±8.37a
4-Terpineol	1.62±0.02c	2.19±0.09a	1.82±0.01b	2.04±0.03b	2.29±0.01a	1.72±0.01c	3.54±0.09c	4.99±0.01a	3.94±0.12b
Neral	1.18±0.01b	1.45±0.09a	1.22±0.05b	1.40±0.09b	1.22±0.02c	1.52±0.01a	1.68±0.01b	2.01±0.10a	1.96±0.03a
α-Terpineol	27.4±1.78c	73.39±8.67a	48.01±0.46b	75.32±5.15b	96.88±3.85a	37.21±0.81c	182.01±5.04c	250.21±0.94a	202.09±3.73b
Geranial	2.01±0.04c	2.31±0.09a	2.19±0.01b	2.87±0.13a	2.29±0.02c	2.44±0.01b	4.30±0.05c	4.99±0.13b	5.18±0.07a
β-Citronellol	2.12±0.01a	2.09±0.05a	2.12±0.02a	2.34±0.04a	2.04±0.01c	2.26±0.01b	5.33±0.03b	4.35±0.11c	6.01±0.11a
γ-geraniol	5.26±0.01a	5.28±0.01a	5.27±0.02a	5.28±0.02a	5.27±0.01a	5.27±0.02a	13.67±0.18b	11.79±0.35c	15.26±0.13a
Nerol	11.57±0.14c	12.98±0.13a	12.46±0.04b	13.96±0.16a	14.02±0.01a	12.27±0.04b	80.47±0.80c	95.24±2.39a	91.19±0.95b

Cis-isogeraniol	0.33±0.03c	1.66±0.03a	1.35±0.02b	1.06±0.01b	1.37±0.03a	0.50±0.01c	3.91±0.04a	3.37±0.02c	3.74±0.01b
Trans-isogeraniol	0.31±0.02c	1.58±0.07a	1.32±0.01b	1.09±0.03b	1.66±0.12a	0.44±0.02c	3.79±0.02b	5.72±0.26a	3.97±0.07b
Geraniol	57.10±1.52c	71.91±1.85a	67.73±0.48b	82.31±2.27a	83.82±0.3a	62.19±0.51b	235.31±1.86b	256.88±4.55a	250.66±3.45a
Geranic acid	39.58±0.27b	39.21±0.21b	40.35±0.07a	40.49±0.05a	38.68±0.19b	38.52±0.01b	98.29±12.25a	66.35±3.30b	79.99±2.60b
Total	390.55±8.46c	485.55±1.98a	420.88±0.99b	605.16±2.94a	553.94±3.73b	527.12±2.51c	1480.57±27.07c	1765.13±5.10a	1652.41±6.33b

Table S2. Changes of bound monoterpenes profiles in Muscat Hamburg grape at six developmental stages.

Bound monoterpenes (μg/L)	E-L 35			E-L 35.5			E-L 36		
	L	M	H	L	M	H	L	M	H
β-Myrcene	14.44±0.05c	16.16±0.18a	15.61±0.15b	18.01±0.40b	20.15±0.05a	17.86±0.06b	22.68±0.77b	26.60±0.30a	21.83±1.15b
Limonene	2.38±0.02b	2.62±0.14a	2.48±0.02ab	3.92±0.16a	3.76±0.10a	3.10±0.05b	6.47±0.12b	7.30±0.25a	5.39±0.62c
Phellandrene	1.93±0.03c	2.12±0.03a	2.02±0.01b	2.69±0.07a	2.71±0.05a	2.38±0.02b	3.57±0.12b	3.85±0.03a	3.61±0.10b
β-trans-Ocimene	12.46±0.03a	12.37±0.45a	12.76±0.01a	13.25±0.09a	13.54±0.40a	13.14±0.02a	14.90±0.07b	15.89±0.25a	14.68±0.04b
γ-Terpinen	4.52±0.02a	4.52±0.07a	4.53±0.03a	4.57±0.01ab	4.60±0.05a	4.54±0.01b	4.54±0.12b	4.75±0.02a	4.62±0.04ab
β-cis-Ocimene	12.79±0.02a	12.57±0.78a	13.28±0.02a	14.11±0.17b	14.78±0.17a	13.88±0.06b	16.83±0.18b	18.55±0.35a	17.05±0.08b
Terpinolen	4.52±0.01a	4.53±0.06a	4.54±0.02a	4.63±0.01a	4.55±0.04b	4.59±0.01ab	4.65±0.19b	5.10±0.06a	4.87±0.01b
Cis rose oxide	5.47±0.01a	5.44±0.09a	5.50±0.01a	5.90±0.03a	5.50±0.05b	5.86±0.01a	6.44±0.09b	6.88±0.04a	6.81±0.05a
Trans rose oxide	nd	nd	nd	0.69±0.15a	nd	0.36±0.02b	2.81±0.01b	3.58±0.04a	3.54±0.20a
Allo-Ocimene	12.08±0.01a	12.08±0.11a	12.16±0.03a	12.26±0.02b	12.40±0.04a	12.29±0.01b	12.28±0.31b	12.88±0.07a	12.62±0.02ab
(E,Z) Allo-Ocimene	12.03±0.01a	12.03±0.07a	12.02±0.02a	12.12±0.01ab	12.16±0.05a	12.07±0.01b	12.15±0.17a	12.33±0.01a	12.23±0.01a
Cis-furan linalool oxide	0.46±0.02c	0.79±0.01a	0.53±0.01b	1.38±0.12b	3.42±0.02a	0.77±0.01c	2.08±0.03b	4.76±0.58a	2.71±0.10b
Trans-furan linalool oxide	0.71±0.01c	1.89±0.01a	0.93±0.03b	1.15±0.16b	3.15±0.05a	1.12±0.17b	1.48±0.03b	3.27±0.58a	1.86±0.04b
Nerol oxide	67.52±0.39a	66.87±6.64a	70.86±0.32a	100.99±2.75a	84.54±5.57b	102.77±1.41a	116.49±1.05c	168.34±6.87a	141.37±0.31b
Citronellal	6.47±0.04c	8.75±0.04a	7.52±0.05b	6.60±0.14b	6.38±0.05b	7.33±0.23a	6.52±0.01c	7.79±0.01a	7.54±0.09b
Linalool	12.19±0.05c	13.37±0.15a	12.53±0.03b	19.12±0.65a	19.79±0.08a	16.46±0.14b	37.35±0.19c	63.18±3.68a	44.35±0.26b
4-Terpineol	6.89±0.02c	8.44±0.13a	7.27±0.05b	6.80±0.02c	7.27±0.06a	6.88±0.01b	6.66±0.10c	7.34±0.15a	6.97±0.03b
Neral	11.78±0.93b	22.72±0.59a	23.38±0.36a	27.96±1.08b	40.83±0.82a	40.10±2.49a	45.82±0.77c	81.48±3.05b	92.28±2.17a
α-Terpineol	41.26±0.36c	60.85±0.69a	47.79±0.48b	39.75±0.49c	47.62±0.45a	40.99±0.18b	41.29±0.12c	52.58±2.65a	45.97±0.38b
Geranial	11.72±0.96c	30.84±0.73a	28.37±1.65b	24.15±1.57c	53.84±0.85a	40.85±2.83b	38.75±0.72c	96.83±9.40a	78.02±2.64b
β-Citronellol	10.25±0.01c	12.59±0.05a	12.10±0.09b	12.02±0.45a	12.58±0.46a	12.66±0.30a	18.01±0.61b	19.80±0.96a	18.98±0.21ab
γ-geraniol	5.27±0.01a	5.27±0.03a	5.28±0.02a	5.28±0.01a	5.29±0.04a	5.30±0.02a	5.28±0.03b	5.35±0.01a	5.33±0.01a
Nerol	58.93±0.17a	58.70±0.65a	58.97±0.90a	77.87±1.39a	74.08±0.06b	70.04±0.38c	97.19±2.14b	100.53±1.13a	97.60±0.30b

Cis-isogeraniol	nd	nd	nd	1.66±0.08a	1.51±0.03a	1.59±0.14a	2.66±0.05b	3.84±0.19a	3.37±0.41a
Trans-isogeraniol	nd	nd	nd	3.24±0.14b	3.83±0.06a	3.37±0.26b	6.99±0.08b	8.95±1.04a	8.21±0.14a
Geraniol	258.42±1.18c	357.89±1.08a	331.31±1.45b	324.51±8.73c	437.61±1.73a	375.85±2.86b	379.89±4.49b	472.48±31.57a	411.7±10.88b
Geranic acid	274.42±7.66c	344.45±1.46b	363.19±4.40a	263.83±10.6c	335.12±4.04a	316.39±9.13b	285.12±1.59b	357.94±39.18a	317.37±10.97ab
Total	849.24±11.40b	1077.87±10.50a	1054.92±16.73a	1008.44±29.29c	1231.02±21.13a	1132.54±17.48b	1198.91±20.68c	1572.18±94.97a	1390.85±28.70b

Data are presented as mean ± standard error (n=3). Different letters indicate significant differences (Duncan's multiple range test, $p \leq 0.05$); nd represents not detected.

Continued Table S2.

Bound monoterpenes (μg/L)	E-L 37			E-L 37.5			E-L 38		
	L	M	H	L	M	H	L	M	H
β-Myrcene	33.53±0.07a	32.10±0.66b	28.85±0.58c	40.50±0.16a	27.79±0.05c	32.32±0.78b	73.21±2.03a	46.55±1.34c	52.08±2.89b
Limonene	9.57±0.51a	9.50±0.23a	8.40±0.60b	12.79±0.24a	11.40±0.04b	9.83±0.36c	27.38±0.74a	15.89±0.73c	17.88±0.86b
Phellandrene	4.92±0.02a	4.69±0.05b	4.59±0.16b	5.99±0.02a	5.33±0.19b	5.03±0.12c	9.59±0.12a	5.99±0.15c	6.77±0.32b
β-trans-Ocimene	17.20±0.13ab	17.69±0.14a	16.70±0.60b	19.98±0.21a	19.37±0.02b	17.87±0.32c	32.38±0.94a	23.28±0.55b	24.23±0.49b
γ-Terpinen	4.81±0.03ab	4.84±0.02a	4.67±0.13b	4.97±0.01a	4.91±0.01b	4.84±0.01c	5.68±0.07a	5.13±0.03b	5.14±0.04b
β-cis-Ocimene	21.53±0.09a	21.37±0.17a	19.98±0.66b	25.46±0.31a	22.45±1.68b	21.73±0.61b	46.59±1.50a	30.99±0.89b	32.21±0.30b
Terpinolen	5.18±0.34a	5.33±0.01a	4.96±0.04a	5.83±0.02a	5.61±0.05b	5.10±0.04c	6.89±0.07a	5.78±0.07c	5.97±0.07b
Cis rose oxide	8.76±0.14a	7.23±0.01b	6.80±0.06c	8.63±0.01a	7.64±0.03b	7.77±0.14b	9.70±0.1a	8.01±0.07c	8.94±0.12b
Trans rose oxide	9.26±0.11a	4.78±0.15b	3.50±0.17c	9.44±0.01a	6.14±0.15c	6.69±0.37b	13.06±0.24a	7.40±0.23c	10.19±0.45b
Allo-Ocimene	13.55±0.09a	13.17±0.02b	12.91±0.06c	13.93±0.01a	13.56±0.01b	13.14±0.07c	18.54±0.19a	15.49±0.14c	15.96±0.14b
(E,Z) Allo-Ocimene	12.47±0.01a	12.49±0.01a	12.38±0.06b	12.69±0.01a	12.63±0.02b	12.50±0.02c	15.33±0.15a	13.81±0.09b	14.00±0.07b
Cis-furan linalool oxide	8.91±0.48a	6.22±0.10b	2.79±0.11c	13.19±0.28a	6.88±0.06b	4.95±0.22c	12.76±0.04a	12.28±0.01c	12.52±0.03b
Trans-furan linalool oxide	3.90±0.52a	2.48±0.15b	1.61±0.07c	3.87±0.02a	2.96±0.20b	1.60±0.13c	11.72±0.01a	11.65±0.01b	11.68±0.01b
Nerol oxide	206.64±3.10a	160.85±1.36b	103.7±0.67c	205.46±1.22a	168.37±0.17b	110.31±3.35c	87.25±1.79a	44.01±0.83c	55.98±2.05b
Citronellal	8.21±0.14a	7.57±0.12b	7.57±0.24b	8.53±0.06a	8.22±0.12b	7.35±0.05c	6.17±0.05a	5.80±0.06c	5.94±0.05b
Linalool	137.92±4.65a	90.31±1.33b	69.59±3.47c	199.98±1.61a	132.33±1.04b	91.91±3.30c	132.76±2.22a	79.87±0.64c	87.42±2.24b
4-Terpineol	7.64±0.08a	7.37±0.01b	6.74±0.02c	7.43±0.02a	7.30±0.02b	6.76±0.01c	0.73±0.03a	0.40±0.01b	0.46±0.06b
Neral	112.90±1.93a	83.91±1.50c	105.00±3.33b	117.58±1.45b	90.23±0.89c	126.53±6.13a	187.75±5.46a	101.01±2.68c	123.03±5.53b
α-Terpineol	62.99±0.21a	54.58±0.28b	42.49±0.48c	68.01±0.57a	56.11±0.26b	45.02±0.58c	53.06±0.39a	44.14±0.11c	46.03±0.50b
Geranial	112.79±2.71a	93.75±7.51b	58.94±1.41c	121.37±6.27a	103.05±0.97b	70.69±4.50c	133.24±3.76a	74.19±5.67c	96.38±3.40b
β-Citronellol	29.28±0.93a	22.44±0.19c	24.31±1.27b	32.76±0.44b	28.6±0.47c	34.32±0.82a	55.03±0.59a	34.25±0.38c	41.56±1.99b
γ-geraniol	5.44±0.03a	5.36±0.02b	5.37±0.01b	5.41±0.01a	5.40±0.02a	5.40±0.01a	105.34±1.10a	53.90±1.01c	68.28±4.27b

Nerol	125.37±0.81a	113.50±1.35b	125.94±0.40a	123.68±0.82b	124.62±0.64b	131.26±1.68a	626.15±0.69a	331.83±7.88c	389.2±29.02b
Cis-isogeraniol	6.24±0.06a	4.56±0.21b	4.41±0.05b	6.60±0.26a	6.13±0.09b	5.40±0.23c	6.78±0.23a	6.35±0.06b	5.51±0.18c
Trans-isogeraniol	17.74±0.10a	12.70±0.11b	12.07±0.42c	20.57±0.73a	16.21±0.55c	18.75±0.89b	4.91±2.60a	4.88±4.66a	5.57±2.83a
Geraniol	551.23±6.77a	486.88±1.21b	481.08±8.08b	518.60±10.75b	548.61±1.32a	467.68±10.43c	734.09±2.82a	465.27±1.32c	496.08±17.95b
Geranic acid	383.43±0.99a	370.69±2.52b	350.23±7.40c	416.11±22.89a	416.48±1.74a	322.48±7.97b	600.49±10.92a	426.22±8.07b	414.04±13.16b
Total	1921.40±10.34 a	1656.36±11.10 b	1525.55±26.82 c	2029.35±30.98 a	1858.33±9.21 b	1587.23±42.72 c	3046.97±44.75 a	1887.14±13.45 c	2071.54±82.71 b

Table S3. Sequencing Data Statistics.

Treatment	Sample ID	Total Reads	Clean reads	Mapped Reads	Average reads	Average mapped reads	GC Content	Q20 (%)	Q30 (%)
H-EL35	H-EL35-1	43441260	21720630	39929418(91.92%)	45343466	41599261	46.77	97.76	93.53
	H-EL35-2	53696502	26848251	49498546(92.18%)	45343466	41599261	46.64	97.64	93.28
	H-EL35-3	38892636	19446318	35369819(90.94%)	45343466	41599261	47.02	97.31	92.6
H-EL36	H-EL36-1	41172052	20586026	37706238(91.58%)	42954600	39307590	47.64	97.28	92.62
	H-EL36-2	44735290	22367645	40711821(91.01%)	42954600	39307590	47.44	96.7	91.44
	H-EL36-3	42956458	21478229	39504711(91.96%)	42954600	39307590	47.6	97.2	92.56
H-EL38	H-EL38-1	44426912	22213456	40765151(91.76%)	42735361.33	39343824.33	47.51	97.28	92.83
	H-EL38-2	43239064	21619532	39951358(92.40%)	42735361.33	39343824.33	47.4	97.38	93.09
	H-EL38-3	40540108	20270054	37314964(92.04%)	42735361.33	39343824.33	47.29	97.11	92.53
L-EL35	L-EL35-1	41839150	20919575	38504639(92.03%)	45636324.67	41691871.67	46.94	97.8	93.58
	L-EL35-2	50089344	25044672	45743722(91.32%)	45636324.67	41691871.67	47.1	97.48	92.98
	L-EL35-3	44980480	22490240	40827254(90.77%)	45636324.67	41691871.67	47.01	97.24	92.45
L-EL36	L-EL36-1	46154210	23077105	42363721(91.79%)	42816332	39224074.67	47.6	97.43	92.98
	L-EL36-2	42520788	21260394	38737902(91.1%)	42816332	39224074.67	47.63	97.18	92.41
	L-EL36-3	39773998	19886999	36570601(91.95%)	42816332	39224074.67	47.43	97.62	93.34
L-EL38	L-EL38-1	45160162	22580081	41003308(90.8%)	42597273.33	38688187.67	47.26	97.06	92.22
	L-EL38-2	40247578	20123789	36795792(91.42%)	42597273.33	38688187.67	47.31	97.03	92.12
	L-EL38-3	42384080	21192040	38265463(90.28%)	42597273.33	38688187.67	47.34	96.83	91.62
M-EL35	M-EL35-1	45414524	22707262	41468410(91.31%)	44733675.33	40490283	47.17	97.78	93.62
	M-EL35-2	43889772	21944886	40451237(92.17%)	44733675.33	40490283	46.73	97.74	93.51
	M-EL35-3	44896730	22448365	39551202(88.09%)	44733675.33	40490283	47.03	97.53	93.05
M-EL36	M-EL36-1	47342896	23671448	43309894(91.48%)	46010918.67	42127556.67	47.7	97.17	92.36
	M-EL36-2	44001640	22000820	40261789(91.5%)	46010918.67	42127556.67	47.67	97.3	92.69
	M-EL36-3	46688220	23344110	42810987(91.7%)	46010918.67	42127556.67	47.43	97.14	92.42

M-EL38	M-EL38-1	44600152	22300076	41173307(92.32%)	44042864.67	40127304	47.52	97.43	93.05
	M-EL38-2	44943596	22471798	41187068(91.64%)	44042864.67	40127304	47.69	97.16	92.38
	M-EL38-3	42584846	21292423	38021537(89.28%)	44042864.67	40127304	47.56	97.77	93.63

Total reads: the number of clean reads, calculated on a single-ended basis. Clean reads: total number of pair-end reads in clean data. Mapped reads: the number of reads mapped to the reference genome and the percentage of reads in clean reads. GC content: clean data GC content, that is, the percentage of bases G and C in the total base in clean data. Q20 and Q30 indicate the ratio of the number of bases in the filtered reads greater than 20 and 30 to the total number of bases, reflecting the quality of the sequencing data.

Table S4. KEGG enrichment analyses of DEGs

ID	Pathway	Number of DEGs	Background number	p-value(<0.05)
	L-EL35 vs M-EL35 vs H-EL35			
ko00940	Phenylpropanoid biosynthesis	23	230	8.61×10^{-7}
ko04141	Protein processing in endoplasmic reticulum	24	271	4.35×10^{-6}
ko00941	Flavonoid biosynthesis	13	100	1.47×10^{-5}
ko00360	Phenylalanine metabolism	11	79	3.55×10^{-5}
ko00945	Stilbenoid, diarylheptanoid and gingerol biosynthesis	6	24	8.11×10^{-5}
ko04075	Plant hormone signal transduction	21	283	0.000237626
ko00910	Nitrogen metabolism	7	46	0.000560949
ko00950	Isoquinoline alkaloid biosynthesis	6	43	0.002219625
ko00908	Zeatin biosynthesis	4	23	0.005518926
ko00905	Brassinosteroid biosynthesis	3	13	0.007213556
ko00130	Ubiquinone and other terpenoid-quinone biosynthesis	6	56	0.008399053
ko00350	Tyrosine metabolism	7	76	0.010266689
ko00942	Anthocyanin biosynthesis	1	1	0.031895554
ko00196	Photosynthesis-antenna proteins	3	18	0.01830882
ko04933	AGE-RAGE signaling pathway in diabetic complications	3	18	0.01830882
ko00906	Carotenoid biosynthesis	4	37	0.029146387
ko04626	Plant-pathogen interaction	16	290	0.022234561
ko00250	Alanine, aspartate and glutamate metabolism	5	56	0.032173934
	L-EL36 vs M-EL36 vs H-EL36			
ko00196	Photosynthesis-antenna proteins	8	18	4.08×10^{-8}
ko00941	Flavonoid biosynthesis	14	100	4.25×10^{-6}
ko04626	Plant-pathogen interaction	23	290	7.41×10^{-5}
ko00940	Phenylpropanoid biosynthesis	19	230	0.000190976
ko00052	Galactose metabolism	9	70	0.000445376
ko00360	Phenylalanine metabolism	9	79	0.001090753
ko00906	Carotenoid biosynthesis	6	37	0.001194022
ko00945	Stilbenoid, diarylheptanoid and gingerol biosynthesis	4	24	0.007320441
ko00040	Pentose and glucuronate interconversions	9	106	0.008098434
ko00261	Monobactam biosynthesis	2	9	0.033579629
ko00908	Zeatin biosynthesis	3	23	0.038723825
ko04141	Protein processing in endoplasmic reticulum	16	271	0.016961087
	L-EL38 vs M-EL38 vs H-EL38			
ko03010	Ribosome	38	372	3.29×10^{-8}
ko00195	Photosynthesis	15	77	2.15×10^{-7}
ko00196	Photosynthesis-antenna proteins	7	18	2.93×10^{-6}
ko04141	Protein processing in endoplasmic reticulum	25	271	5.29×10^{-5}
ko03060	Protein export	8	53	0.000973491
ko00941	Flavonoid biosynthesis	11	100	0.001774116
ko00190	Oxidative phosphorylation	16	203	0.005992736

ko00942	Anthocyanin biosynthesis	1	1	0.039332342
ko00360	Phenylalanine metabolism	8	79	0.011945126
ko03020	RNA polymerase	6	65	0.041566799

KEGG enrichment analyses of DEGs. Pathway: name of KEGG pathway. Ko: KEGG path id. P-value: statistical significance of enrichment. The smaller the P-value, the higher the enrichment degree, *P*-value≤0.05.

Table S5. Number of genes in each WGCNA module

Module name	Gene number
MEblack	137
MEblue	1932
MEbrown	492
MEgreen	162
MEgreenyellow	67
MEmagenta	112
MEpink	118
MEpurple	67
MEred	150
MEtan	55
